# Practical Lessons in the Nature and Treatment of the Affections Produced by the Contagious Diseases

**Published:** 1872-12

**Authors:** 


*Practical Lessons in the Nature and Treatment of the Affections Produced by the Contagious Diseases, with an Account of the Primary Syphilitic Poison and of its Communicability.* By JOHN MORGAN, A. M., M. D., University of Dublin. Philadelphia: J. B. Lippincott & Co., 1872; Buffalo: H. H. Otis.

This work is evidently written by one who has made a careful study of syphilis and the syphilitic poison in most of its forms. He presents to the reader some original ideas which differ somewhat from the already established views of the profession, but these exceptional cases are few, and careful study and observation will, in some instances, we have no doubt, convince of their correctness.

The author lays considerable stress upon the fact that syphilis may be contracted by inoculation with the vaginal discharge, where no local sore exists. The work is for the most part well written, and is amply illustrated. We welcome it as adding other facts to the fast accumulating mass of evidence concerning syphilis, its nature and treatment. Our author is a believer in syphilization, and quotes cases from our great American authority, Prof. Bumstead, of New York. Many of his teachings are to be accepted with some reservation on the part of the reader, and some allowance made for observations made almost exclusively in hospital practice.

